# Re: Daniela E. Oprea-Lagera, Tessa van Elst, Shafak Aluwini, et al. How Does Routine Prostate-specific Membrane Antigen Positron Emission Tomography/Computed Tomography Modify the Current Management of Prostate Cancer? A Multidisciplinary View. Eur Urol Open Sci 2025; 75: 69–79

**DOI:** 10.1016/j.euros.2025.05.017

**Published:** 2025-09-26

**Authors:** Birhan Demirhan, Aysenur Elmali, Cem Onal

**Affiliations:** a Division of Radiation Oncology, Iskenderun Gelisim Hospital, Hatay, Turkey; b Department of Radiation Oncology, Başkent University Faculty of Medicine, Ankara, Turkey; c Department of Radiation Oncology, Adana Dr. Turgut Noyan Research and Treatment Center, Başkent University Faculty of Medicine, Adana, Turkey

We commend Oprea-Lager et al [1] for their comprehensive national consensus study evaluating the clinical impact of prostate-specific membrane antigen (PSMA) positron emission tomography/computed tomography (PET/CT) on prostate cancer management in The Netherlands. As the integration of next-generation imaging (NGI) technologies into clinical practice increases, such multidisciplinary efforts are timely and informative. However, several methodological limitations warrant discussion to prevent overinterpretation of consensus as a substitute for evidence-based practice.

The study explored treatment intensification and deintensification on the basis of PSMA PET/CT findings, yet there is still a lack of prospective data demonstrating that such imaging-guided changes improve clinical outcomes. Although PSMA PET/CT surpasses conventional imaging in sensitivity and specificity, modification of treatment strategies—particularly when deviating from frameworks such as CHAARTED [2] that are based on conventional imaging—remains speculative without robust clinical correlation [[3], [4], [5]]. Adoption of PSMA PET/CT as the sole determinant for altering systemic therapy or radiotherapy strategies risks the promotion of unvalidated shifts in clinical practice. Until prospective randomized trials establish the impact of PSMA-guided treatment decisions on survival or toxicity outcomes, caution is warranted in extrapolating these findings to routine care.

A major limitation lies in the absence of standardized and validated criteria for defining metastatic burden—specifically, for distinguishing low- versus high-volume disease—on PSMA PET/CT. Although the panel proposed the use of lesion count and the axial distribution as surrogate markers, these proxies have not been rigorously validated and are susceptible to significant interobserver variability. Moreover, the impact of equivocal or borderline findings on PSMA PET/CT interpretations was not systematically accounted for, which could potentially compromise the reproducibility and reliability of volume-based stratification [6]. This undermines consistency in clinical decision-making and highlights the need for uniform PSMA-specific stratification guidelines.

Furthermore, while the authors acknowledge that pivotal trials such as CHAARTED, LATITUDE, and PEACE-1 were based on conventional imaging, the study does not address how PSMA-based reclassification might misalign patients with trial-defined eligibility criteria [2,7,8]. Specifically, the potential for upstaging or a change in disease volume categorization using PSMA imaging may lead to misclassification of patient eligibility for evidence-based treatment intensification. This discrepancy undermines the extrapolation of trial-derived outcomes to patients restaged via NGI and introduces the risk of guideline drift in the absence of prospective validation. Without high-level evidence confirming the clinical benefit of PSMA-informed treatment modifications, such practice shifts remain premature and potentially misleading.

In summary, while the panel’s findings provide valuable insight into evolving clinical perspectives, they should not be interpreted as prescriptive guidance. Until prospective trials confirm the clinical benefit of PSMA-informed decisions, such imaging should be used judiciously. Standardization efforts—such as the PROMISE V2 framework—are essential to ensure that imaging precision translates into meaningful treatment outcomes [9].

  ***Conflict s of interest*:** The authors have nothing to disclose.

## References

[1] Oprea-Lager D.E., van Elst T., Aluwini S. (2025). How does routine prostate-specific membrane antigen positron emission tomography/computed tomography modify the current management of prostate cancer? A multidisciplinary view. Eur Urol Open Sci.

[2] Sweeney C.J., Chen Y.H., Carducci M. (2015). Chemohormonal therapy in metastatic hormone-sensitive prostate cancer. N Engl J Med.

[3] Hofman M.S., Lawrentschuk N., Francis R.J. (2020). Prostate-specific membrane antigen PET-CT in patients with high-risk prostate cancer before curative-intent surgery or radiotherapy (proPSMA): a prospective, randomised, multicentre study. Lancet.

[4] Calais J., Czernin J., Cao M. (2018). ^68^Ga-PSMA-11 PET/CT mapping of prostate cancer biochemical recurrence after radical prostatectomy in 270 patients with a PSA level of less than 1.0 ng/ml: impact on salvage radiotherapy planning. J Nucl Med.

[5] Onal C., Guler O.C., Erpolat P. (2024). Evaluation of treatment outcomes of prostate cancer patients with lymph node metastasis treated with definitive radiotherapy: comparative analysis of PSMA PET/CT and conventional imaging. Clin Nucl Med.

[6] Seifert R., Gafita A., Telli T. (2024). Standardized PSMA-PET imaging of advanced prostate cancer. Semin Nucl Med.

[7] Fizazi K., Tran N., Fein L. (2017). Abiraterone plus prednisone in metastatic, castration-sensitive prostate cancer. N Engl J Med.

[8] Fizazi K., Foulon S., Carles J. (2022). Abiraterone plus prednisone added to androgen deprivation therapy and docetaxel in de novo metastatic castration-sensitive prostate cancer (PEACE-1): a multicentre, open-label, randomised, phase 3 study with a 2 × 2 factorial design. Lancet.

[9] Eiber M., Herrmann K., Calais J. (2018). Prostate Cancer Molecular Imaging Standardized Evaluation (PROMISE): proposed miTNM classification for the interpretation of PSMA-ligand PET/CT. J Nucl Med.

